# Feasibility of Using Wideband Microwave System for Non-Invasive Detection and Monitoring of Pulmonary Oedema

**DOI:** 10.1038/srep14047

**Published:** 2015-09-14

**Authors:** S. Ahdi Rezaeieh, A. Zamani, K. S. Bialkowski, A. Mahmoud, A. M. Abbosh

**Affiliations:** 1 School of ITEE, The University of Queensland, St Lucia, 4072, Brisbane, Australia; 2 School of Medicine, Griffith University, Gold Coast, 4215, Australia

## Abstract

Pulmonary oedema is a common manifestation of various fatal diseases that can be caused by cardiac or non-cardiac syndromes. The accumulated fluid has a considerably higher dielectric constant compared to lungs’ tissues, and can thus be detected using microwave techniques. Therefore, a non-invasive microwave system for the early detection of pulmonary oedema is presented. It employs a platform in the form of foam-based bed that contains two linear arrays of wideband antennas covering the band 0.7–1 GHz. The platform is designed such that during the tests, the subject lays on the bed with the back of the torso facing the antenna arrays. The antennas are controlled using a switching network that is connected to a compact network analyzer. A novel frequency-based imaging algorithm is used to process the recorded signals and generate an image of the torso showing any accumulated fluids in the lungs. The system is verified on an artificial torso phantom, and animal organs. As a feasibility study, preclinical tests are conducted on healthy subjects to determinate the type of obtained images, the statistics and threshold levels of their intensity to differentiate between healthy and unhealthy subjects.

Pulmonary oedema is the common manifestation of various diseases, such as hypertension, pulmonary infections, acute heart failure and respiratory distresses, that are among the leading causes of fatality worldwide^1^. As an example, the early pulmonary manifestation of the acute phase of decompensated heart failure is the congestion of the vascular bed due to increased capillary hydrostatic pressure. As the pressure further increases and the lymphatic drainage is overloaded, the fluid begins to accumulate in the interstitium, which is around the blood vessels, airways and interlobular septa^2^. Through invasive monitoring systems of pulmonary pressure, it has been shown that acute heart failure has a relatively long incubation period, up to two weeks, during which pulmonary oedema can be identified before the appearance of clinical signs^3^,^4^. Therefore, if that accumulated fluid is detected and monitored early when it is small in volume, it allows a timely treatment to avoid further complications such as volume overload and worsening cardiac function. Previous research and current guidelines indicate the importance of monitoring volume overload and timely treatment to reduce hospitalization^5^,^6^,^7^.

Computer tomography (CT) and chest X-ray are the most widely used diagnostic tools for lungs’ fluid detection. However, their ionization nature and lack of sensitivity prevents their use as a long-term monitoring tool. As a result, microwave based medical diagnostic systems as safe, affordable and portable detection and monitoring tools are being developed. The use of microwaves for lungs’ fluid detection was first investigated in 1973 when it was shown that the node of the standing wave ratio change when encountering variable water content within the lungs^8^ due to the variation in the effective dielectric constant of the lungs. This is the principle of microwave diagnostic systems that is widely utilized to detect vital signs^9^,^10^,^11^,^12^ and cancerous tumors, which inherently have high water contents^13^,^14^. The radiometric technique is another approach, which measures radiation from the human body to determine the fluid changes inside the lungs^15^. Later on, long-term phase variations were considered as an appropriate approach to monitor changes in fluid levels^16^. Estimation of lungs tissues’ electrical properties and lungs’ imaging are the most recent developments aiming to detect any fluid accumulation in the lungs by monitoring the changes in the effective dielectric properties of the lungs or locating the strong scatterers within the lungs, respectively^17^,^18^.

The design and realization of a non-invasive and portable microwave system, which has an antenna array platform embedded inside a bed and can be easily used in preclinical and clinical trials, is proposed in this paper. This system aims at detecting and monitoring small amounts of fluid inside the lungs that can be caused by various diseases. This system will provide preliminary data for medical staff to pursue further investigations to define the exact cause of the oedema. Additionally, it can be used to monitoring the progress of the disease or treatment in pre-hospitalization or rehabilitation process, respectively. The system consists of a hardware unit for data acquisition and software unit for processing and image formation. The hardware unit comprises of two linear antenna arrays, a switching network, a portable vector network analyzer (VNA) and a laptop which includes the software used for control, processing and image formation. One of the main constrains that have hindered the design of realistic platforms for microwave-based diagnostic systems for pulmonary oedema is the size of the antennas. In order to provide the required penetration of the signal into the torso area while offering a reasonable image resolution, the frequency band needed for this application is the low microwave spectrum within 0.7–1 GHz^18^. Several antennas have been designed to operate at that band^18^,^19^,^20^. However, they either have a large size^18^,^19^,^21^, or require a matching medium^20^. In order to overcome these problems, a three dimensional structure that employs a combination of loop, monopole, and parasitic patch antennas is proposed in this paper. The achieved miniaturized size enables placing large number of antenna elements in the proposed platform for better image resolution and detection sensitivity.

To monitor the changes inside the lungs and detect fluid accumulation, a frequency domain imaging algorithm, which utilizes backscattered signals from the torso to map the scatterers inside the lungs, is proposed as part of the system. The algorithm is based on the fact that the relation between the measured microwave signals by the utilized antenna array with the scattered signals inside the torso is according to the first order Bessel function of first type. Due to the fact that microwave signals experience higher reflection from fluid compared to the surrounding lungs’ tissues, the obtained image shows higher scattered electromagnetic field intensity at the location of the fluid. The presented algorithm does not need solving ill-posed problems of wave path estimation, which is one of the main drawbacks of time-domain techniques. The operation of the designed system is tested on an artificial phantom that has the precise anatomical size of an average human torso and includes cardiovascular organs such as lungs and heart as well as ribs and fat. The obtained results are further verified using lamb lungs, which have similar dielectric properties to human lungs. The experimental results indicate that the proposed system is capable of detecting fluid accumulation inside the lungs as low as 1 mL. Finally, preclinical tests are conducted on five healthy volunteers to establish a threshold level of the expected level of predicted scattered signals inside the lungs. The results of the study, which is conducted in an uncontrolled environment with possible effects of subject movement and breathing, and noise, suggest that the obtained threshold has a certain value for all those cases with 10% variation. That threshold is needed in the future trials on subjects with and without pulmonary oedema.

## The proposed pre-clinical system

**Figure 1:**
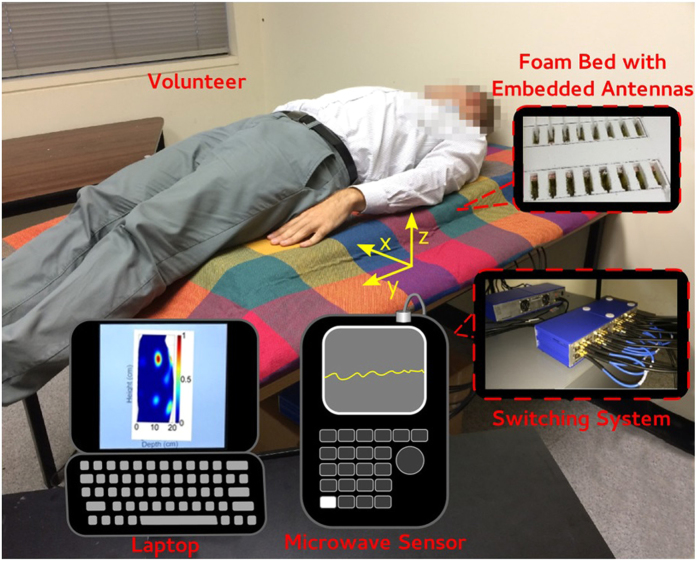
Proposed preclinical microwave imaging system of the torso for pulmonary oedema detection.

The proposed system for pulmonary oedema detection is depicted in Fig. 1. The utilized antenna arrays are designed and fitted into a series of slots that are cut and trimmed from a radio frequency (RF) transparent foam block, which acts as the base of the bed (platform). As depicted in Fig. 1, during tests, the subjects lay on their back so that the antenna array faces the rear side of the torso that provides similar structures in male and female subjects. In order to scan the torso area at the central lines of the two lungs, the platform comprises of two sets of linear antenna arrays. The two arrays are located at a distance of 11 cm from each other, which equals the distance between the center of the left and right side lungs of an average human being. The two antenna arrays are connected to a switching network formed using USB-8SPDT-A18 switches^22^. A portable Agilent N9923A FieldFox RF vector network analyzer (VNA)^23^ is used as the microwave transceiver of the system. It generates the required frequency-domain microwave signals, transmits them via the antenna arrays to the torso, captures the received back-scattered signals from the torso, and sends them as digital data to a laptop. These data are then processed by that laptop using the proposed frequency domain based algorithm, which is also used to generate an image of the scattered field inside the torso.

## Antenna Design

Antennas are one of the key elements of any microwave based medical diagnostic system and therefore, the success of that system significantly depends on the performance of those antennas. A wide operating bandwidth and unidirectional radiation are the two major requirements for microwave imaging systems to obtain high resolution images and eliminate surrounding environment’s effects, respectively^18^. As depicted in Fig. 1, the proposed system is intended to have two linear arrays to scan the torso area and therefore, the size of the antennas on the x-y plane, defines the maximum number of usable antennas at the constrained area of the torso. Several techniques, such as metamaterials and three dimensional (3-D) structures have been proposed to reduce the size of the planar antennas. However, despite their excellent size reduction abilities, their bands are usually limited. On the other hand, planar structures such as tapered slot or quasi-Yagi antennas provide simple configurations and wide operating bandwidths; however, they have large profiles, and thus significant mutual coupling occurs between the antenna elements when used to scan the limited torso space. Only recently a technique based on folding provided a way to successfully achieve wide band performance^24^,^25^. Nevertheless, the structure of those antennas reveals that the main size reduction occurs only in the x-z and y-z planes. The previously utilized 3-D miniaturization techniques are not effective on the main radiating plane (x-y), which defines the number of antennas to be used to scan the torso, mainly due to the fact that locating the radiating plates in close proximity to each other disturbs the high current concentration areas on the feeding line and the ground plane resulting in the formation of a large capacitive/inductive reactance in the input impedance that makes the impedance matching an extremely challenging task.

**Figure 2:**
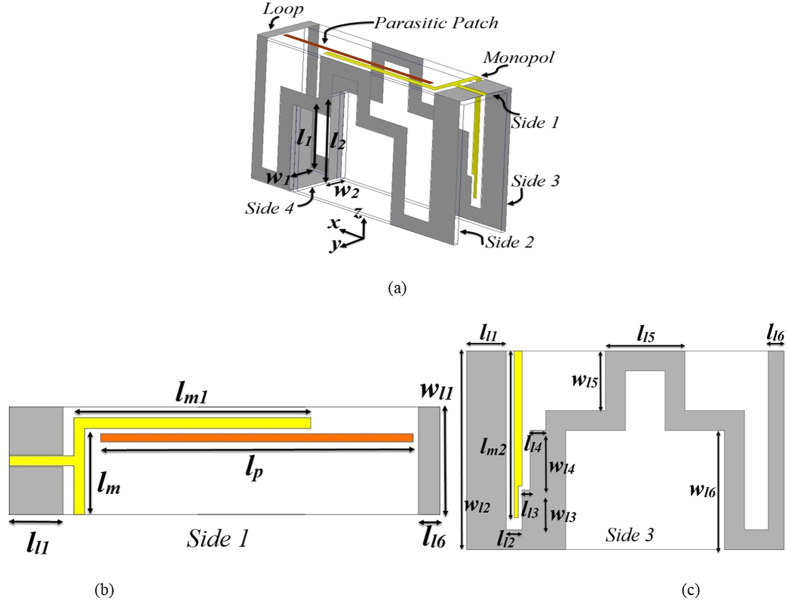
Geometry details of the optimum design in (**a**) 3-D configuration, (**b**) top view (Side 1) and (**c**) right side view (Side 3).

To overcome the above stated problems, a series of approaches are suggested in this paper to build the antenna depicted in Fig. 2. The structure is designed on four FR4 substrates with 1.6 mm thicknesses that have a relative permittivity of 4.4 and loss tangent of 0.02. The printed substrates are connected together to form a three-dimensional structure with the help of solder and copper tape from the outer side of the structure. The antenna is comprised of a meandered loop and an L-shaped monopole antenna in addition to a parasitic patch. The simple loop antenna is selected as the base structure of the design to enhance the impedance matching and directivity. The length of a conventional loop is proportional to one wavelength at the resonant frequency. However, to compensate for the intense coupling between the two vertical sides of the design that causes a shift in the resonance to higher frequencies^21^, the perimeter of the loop is selected as one wavelength at the low frequency of 600 MHz, which is lower than the targeted low frequency of around 0.7 GHz. To accommodate such a large perimeter in a confined structure, a meandering technique is utilized^26^. Besides, two rectangles with dimensions of *w*_*1*_ × *l*_*1*_ and *w*_*2*_ × *l*_*2*_ that are printed on the same substrate with similar thickness are connected to the top portion of the loop structure to alleviate the high capacitive reactance of the input impedance. The top portions of the sides 2 and 3 are connected to each other using a rectangle patch with dimensions of *w*_*l1*_ × *l*_*l1*_ to alter the surface current circulation and consequently improve the directivity.

**Figure 3:**
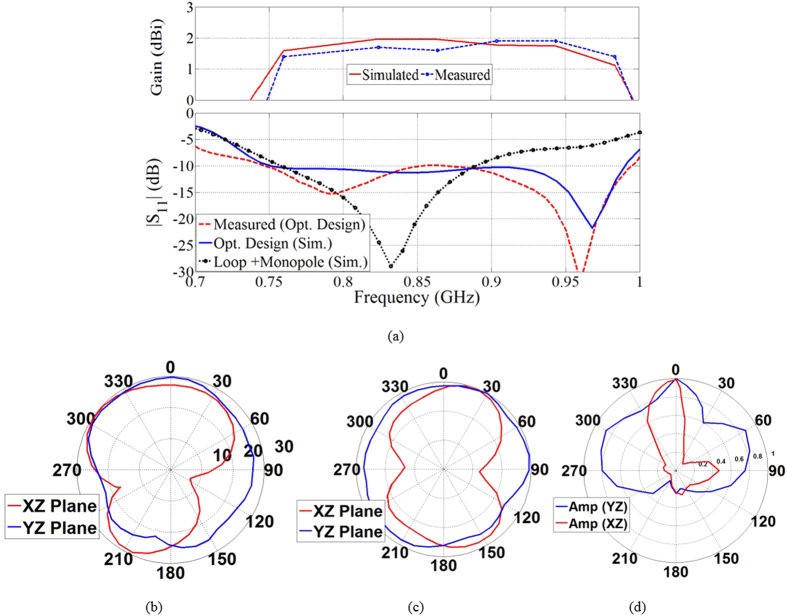
(**a**) Simulated and measured S_11_ performance and gain of the optimum design and S_11_ performance of the antenna during the evolution process. Far-field radiation pattern of the optimum antenna at (**b**) 800 MHz and (**c**) 950 MHz. (**d**) Time domain near-field of the antenna across the operating bandwidth.

To compensate for the narrow operating bandwidth of the loop structure, an L-shaped monopole element is used to feed the whole structure. The monopole is fed using a coplanar waveguide (CPW) line with a length of 12 mm and width of 1.8 mm, which is separated by a gap of 0.2 mm from the loop structure for a 50 Ω port impedance. The lengths of the monopole (*lm* + *l*_*m1*_ + *l*_*m2*_) is selected as about quarter wavelength at about the center frequency, which is around 850 MHz assuming a band from 700 MHz to 1000 MHz. As depicted in Fig. 3(a), the latter modification expands the 10 dB return loss of the antenna to 760–880 MHz. The staircase structure on the monopole and loop antenna at side 3 is used for impedance transformation and tuning purposes^25^. To fulfill the band requirements of a lung fluid detection system, a half wavelength resonating parasitic patch at the upper frequency of 1000 MHz, with 1.5 mm width and length of *l*_*p*_, is located at a distance of 1.5 mm from the monopole structure. As a result of proximity coupling, the parasitic patch resonates at 960 MHz, which is slightly less than the targeted value of 1000 MHz, and increases the operating bandwidth of the antenna to 750–985 MHz, which is proportional to fractional bandwidth of 27% as verified by measured results. From the same plot, it can be realized that the antenna has a moderate peak gain of 1.9 dBi in +z direction. It should be noted that the moderate gain of the antenna is the best outcome of the highly miniaturized antenna size.

One of the main requirements of the antenna to be used in fluid detection is a unidirectional radiation, i.e. in the forward direction or +z in this case. In the proposed structure, a unidirectional radiation is achieved by the 3-D structure of the loop antenna. As depicted in Fig. 3(b,c), the measured far field radiation patterns of the antenna at the two sample frequencies of 800 MHz and 950 MHz reveals that the antenna has a unidirectional radiation pattern with a reasonable average front to back ratio of 4 dB in the far-field measurements that is considered satisfactory for medical imaging applications^27^. The obtained directivity is the result of phase variations of the antenna’s surface currents, which tend to cancel each other at the center of the structure when it is folded^28^.

Due to the fact that in the proposed system, the antenna operates at a short distance from the human torso, the near field radiation of the antenna was calculated using the time domain solver of CST microwave studio software. The amplitude of the fields are calculated using 30 co-polarized electric field probes that are located around the perimeter of the antenna with 12° intervals in x-z and y-z planes at a distance of 2 cm with respect to the antenna’s surface. The obtained results are depicted in Fig. 3(d). The higher value of the field in the y-z plane of the main lobe of the pattern is due to the fact that the probes are in immediate proximity to the radiating elements on sides 2 and 3 and therefore receive higher values compared to the x-z plane ones. Nevertheless, the calculated time domain front to back ratio of the fields show a value of 4:1 confirming the directive performance of the antenna across its operating band^24^. Additionally, as the antenna is intended to be used in human trials, the specific absorption rate (SAR) in a human body using this antenna was calculated using the simulator HFSS. The antenna was located at a distance of 2 cm from a numerical three-dimensional torso model with 2 mm resolution. As is general practice, the SAR value was calculated on 10 grams of tissue^20^. Using the same microwave power level of the suggested system, i.e. 1 mW (0 dBm), the calculated SAR value was found to be 0.03 (W/Kg), which is well below the specified safe limit for medical applications^29^. With the optimum values presented in Table 1, the proposed antenna achieves a compact size of 0.05 λ × 0.2 λ × 0.12 λ (where λ is the wavelength at the lowest operating frequency band), which is around half the size of the most compact antennas designed to operate at the same bandwidth on x-y plane^24^,^25^,^26^.

## Imaging Process

The imaging technique consists of preprocessing techniques to eliminate the noise and background reflections from the recorded raw data by the antenna array and an image reconstruction algorithm, which generates an image to help in the pulmonary oedema detection.

If an electromagnetic wave is sent towards the human body, a large portion of that wave is reflected back due to the high permittivity of the skin. The strong reflection represents clutter that can mask the target, especially if it is small at the early stages of pulmonary oedema. Therefore, a proper preprocessing technique is required to remove or significantly mitigate the skin reflections.

Considering the skin as a dielectric slab with a dielectric constant of *ε*_*r*_, the reflection transfer function of the skin can be expressed by^30^:


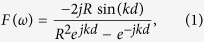


where *d* is the thickness of the skin, 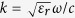 is the wavenumber, *c* is the wave speed in vacuum and *R* is:


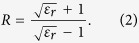


The reflected signal from a layer can be calculated by multiplying the incident wave by (1), which can be strong enough to mask the backscattered wave generated by the target. Therefore, the reflected signal should be removed before applying the imaging method. Although the aforementioned reflection transfer function doesn’t provide the reflected signal from a multi-layered media, it shows that the reflected wave from each layer depends on the layer thickness and dielectric properties and the frequency of the incident wave. By assuming a uniform skin thickness on the torso and the antennas having being the same distance away from the skin, the reflected wave behaves similarly at each antenna. In this case, the reflection can be removed by subtracting the average value of the received electric field (*E*_*avg*_) in an antenna array from the value of each antenna of the array^31^. To do so, the electric field (*E*_*meas*_) is extracted from the measured S-parameters (*S*_*meas*_), for each of the antennas in the array and the average value over the array is subtracted:


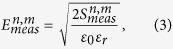


and


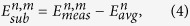


where, *n* and *m* represent the antenna number and frequency step, respectively, and *ε*_*0*_ is the permittivity of free space. Applying the average subtraction method for all the antennas and frequency steps provides *N*_*p*_ × *N*_*f*_ values of subtracted electric field (*E*_*sub*_) at each antenna array.

When boundary (skin) reflections are removed, the difference in the dielectric properties of different points within the imaged region can be extracted from the inequality of electric fields at different antenna positions and frequencies. To that end, the imaged area is discretized into square cells and the *N*_*p*_ × *N*_*f*_ calculated fields at each cell are added together to show the distributions of dielectric contrasts within the imaged domain^32^.

The torso is a heterogeneous medium that consists of many tissues with different dielectric properties. Thus, to emphasize the larger intensities, the subtracted field is squared, and then normalized using the square of the incident field.


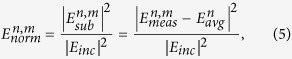


where *E*_*inc*_ is the known incident field and is constant for all the antennas. This has the effect of reducing the intensity of the low intensity fields scattered which may otherwise could be mistaken for false targets.

**Figure 4:**
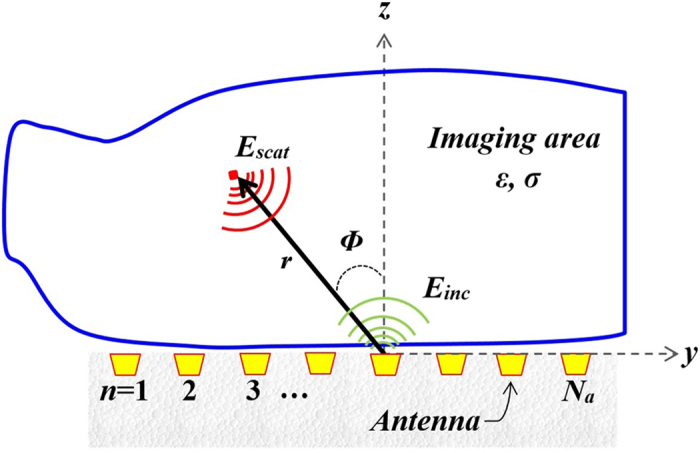
Diagram of the utilized torso imaging domain.

Maxwell’s equations can be applied to the normalized received field (*E*_*norm*_) to obtain a map of scatterers within the imaged domain. As depicted in Fig. 4, each antenna of a *N*_*a*_-element flat array transmits an incident field into the imaging area (a cross section of the torso) and receives the backscattered field. The scattered electric field from each point (*x*, *y*) inside the imaged area is calculated using Bessel function of the first kind of order zero *J*_*1*_(.)^33^:


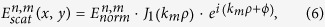


where *k*_*m*_ is the wavenumber of *m*-th frequency step and (*ρ*, *Φ*) represent the distance and the angle of each point within the imaged domain from the antenna position. Applying the summation process to the estimated scattered field, over all the antennas (*n* = 1 to *N*_*a*_) and frequency steps (*m* = 1 to *N*_*f*_) provides the location and intensity of high contrasts of dielectric constants inside the torso for each array:


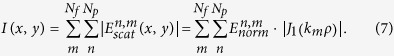


In order to obtain a threshold for healthy and unhealthy cases, the *I*(*x, y*) values of the left and right side arrays are subtracted from each other:


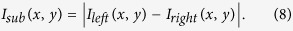


Due to the fact that the structure of the left and right side lungs have similarities^18^, the subtraction process highlights any changes that may have occurred due to the presence of a target (fluid inside the lungs).

In the imaging process, different antennas of a mono-static array are taken into account simultaneously and the electromagnetic equations are solved in frequency domain to not only show the significant scatters’ location, but also accelerate the imaging procedure. Additionally, the effect of the system delays and the need for solving ill-posed problems of wave path estimation, which are the major problems in time-domain techniques, are removed by performing the calculations in the frequency domain.

## Experimental results and discussion

**Figure 5:**
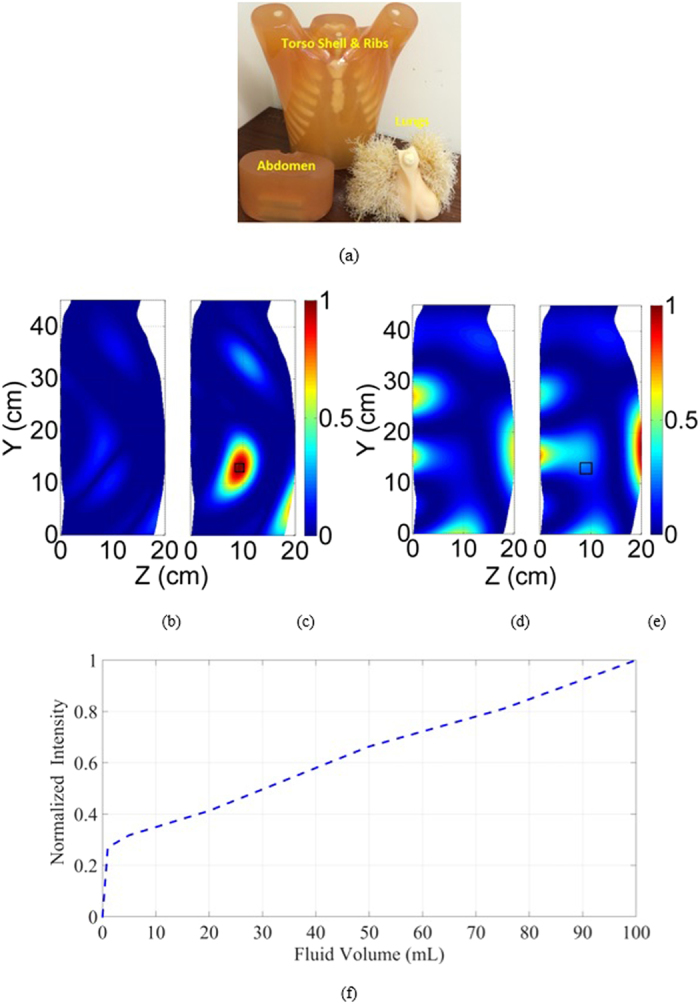
(**a**) The artificial phantom used in the tests. Images from using 8 × 2 array configuration on artificial phantom: (**b**) healthy and (**c**) unhealthy (1 mL inserted water). Images from using 4 × 2 array configuration: (**d**) healthy and (**e**) unhealthy (1 mL inserted water). (**f**) Variations of the intensity of differential scattered field with the increase of water volume using 8 × 2 array (normalized with respect to the field value at 100 mL fluid volume).

Several experiments were conducted to verify the performance of the presented system. In one of the experiments, eight antennas were fitted in each array with a spacing of 3 cm and reflection coefficient of −6 dB. The system is tested on the artificial torso phantom (Fig. 5a) that has realistic anatomical size and includes fat, muscle, ribs, lungs, heart and abdomen block with reasonably realistic dielectric properties. Although the phantom does not include the blood circulation system or breathing functions, it provides a much more complex environment compared to recently reported studies^16^,^17^, and hence provides a reasonable estimate of realistic case. A healthy case was firstly considered when there is no fluid (water) inside the lungs of the phantom. The signals from both arrays are recorded. To emulate a pulmonary oedema case, various volumes of water were inserted into the lungs of the phantom. In another experiment, the same process is repeated but using four antennas in each array that are located in a 6 cm distance to see whether it is viable to use small number of antennas in the platform. The generated images using the proposed algorithm are depicted in Fig. 5. The presented results are the absolute values that are obtained after subtracting the scattering profiles of right and left sides from each other (8). The obtained subtracted images for healthy and unhealthy cases in the first scenario are shown in Fig. 5(b,c). As it can be realized, the maximum normalized intensity of the scattered field inside the torso is around 90% higher than the healthy one for 1 mL water content located at the lower portion of the lung (black rectangle). This detectable volume of fluid is four times better (lower) than the least detectable amount by recent systems^25^. The obtained results for the second setting with only four antenna elements on each array are also depicted in Fig. 5(d,e) for healthy and unhealthy cases with 1 mL accumulated water. As it can be seen, the obtained images do not enable an accurate detection of the accumulated water. The successive experimental results revealed that the system with small number of antenna elements is not able to detect water contents lower than 4 mL using this experimental setup. Therefore, the first configuration with larger number of antennas provides better results and therefore, selected for further tests. To evaluate the monitoring ability of the system in defining different volumes of accumulated fluid, various amounts of water were inserted into the lungs and the reflected signals were assessed. As can be seen from Fig. 5(f), the intensity of the obtained scattered field increases linearly with the amount of water inside the lungs, and hence can be used as a measure of progress of the fluid accumulation during different stages of the oedema. In this figure, the zero volume represents a healthy case and the intensity value is normalized with respect to the value of the differential field at 100 mL fluid volume.

## Methods

**Figure 6:**
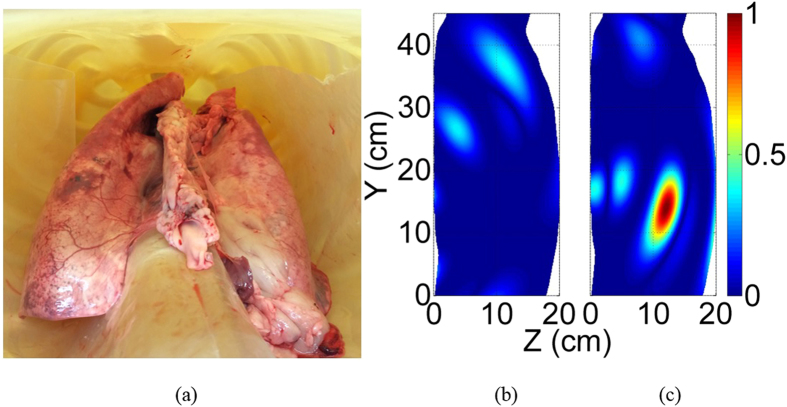
(**a**) The phantom used in the tests: Lamb lungs inside an artificial torso. Images from using 8 × 2 array configuration on that phantom: (**b**) healthy lamp and (**c**) lamb with 1 mL injected water.

To verify the obtained results in a more realistic scenario, the artificial lungs were replaced with a pair of lamb lungs depicted in Fig. 6(a). The dielectric properties of the lungs were measured using Agilent 85070E dielectric probe and an average dielectric constant of 49, which is very close to that of a deflated human lung, is obtained across the operating band of the system. The experiments were conducted in accordance with the guidelines and protocols which were approved by The University of Queensland’s risk management procedure. To emulate pulmonary oedema, 1 mL of water was injected into the lower side of the left side lung. The obtained results depicted in Fig. 6(b,c) validate the capability of the system. The imaged profile of the unhealthy case is stronger at the location of the water with a ratio of 2.5:1.

**Figure 7:**
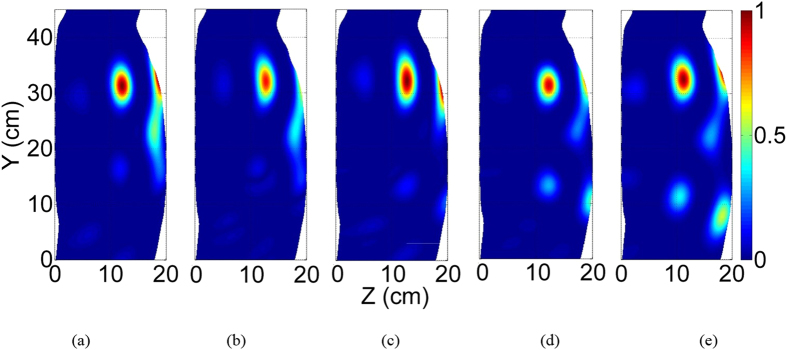
Example of the obtained torso images of healthy volunteers. The high intensity area indicates the location of the heart.

**Figure 8:**
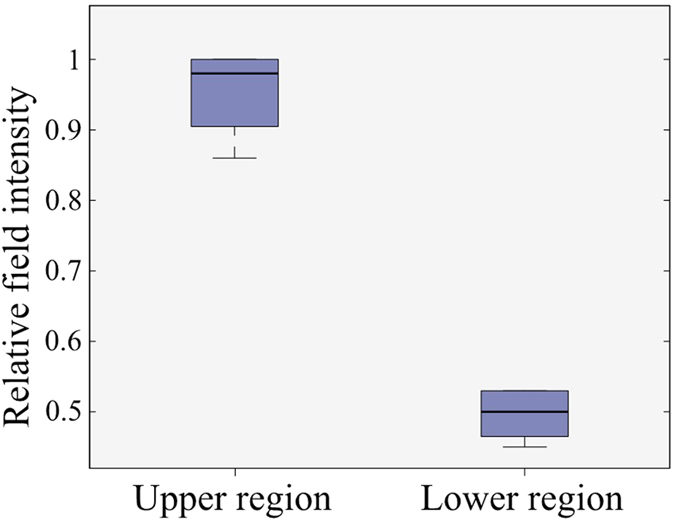
Statistical properties of the field intensity over the subjects.

Having the reliability of the system attested, a series of preclinical trials were conducted on healthy volunteers. As a common procedure in medical diagnostic applications^14^,^34^, these studies were conducted with the aim of investigating the possibility of building a database for healthy and unhealthy people with different physical sizes. The main goal of the study is to investigate whether the obtained intensity levels for the scattering profile of the torso are in a confined range or not. A set of tests were performed, all in accordance with the guidelines and protocols which were approved by the Ethics Committee of The University of Queensland (Australia). An informed consent was obtained from all subjects involved in the study. All measurements followed the previously explained process that is in accordance with the regulations and guidelines approved by the Ethics Committee. The experiments were performed under an unrestrained environment where the effects of subject’s movement and breathing, and external noise are included in the measurements. An example of the images obtained are depicted in Fig. 7, with the statistical properties of the upper and lower region of the image shown in Fig. 8. The images are normalized with respect to the maximum field intensity obtained over all of the volunteers. In each image, there is a point of high scattering located in the upper region. From to the location of this scatterer and the known dielectric properties of the torso’s organs in the used frequencies, this value can be attributed to the heart, which has a high effective dielectric constant due to the blood inside the heart. This indicates the possibility of detecting strong scatterrers inside the torso. In the presence of accumulated water in unhealthy situations, it is expected that the obtained images will show the water inside the lungs as the highest scatterer, because of its higher dielectric constant than the effective dielectric constant of the combination of heart and blood. From the obtained results, the variation of the normalized subtracted electromagnetic field intensity inside the torso is in a confined range with only 10% variation in all regions of the torso. Although preliminary, the obtained results encourages the possibility of building a global database that can be used as a definite range for healthy people and therefore, can be used as a threshold to detect unhealthy cases.

## Conclusion

A preclinical platform for early stage detection and monitoring of pulmonary oedema has been presented. The proposed system consists of two planar antenna arrays that are embedded inside a foam-based bed to provide a convenient and portable platform for clinical trials, a switching network, microwave transceiver and laptop for control, signal processing and image formation. The utilized antennas have a compact size to facilitate using large number of elements for accurate detection. To that end, a combination of meander loop, monopole and parasitic patch antennas is employed in a three dimensional structure. The proposed antenna was verified to have wide operating bandwidth and unidirectional radiation both in near and far fields. Additionally, a frequency domain based imaging algorithm has been presented to map the scattering profile of the torso area for each lung. The proposed system was successfully tested on artificial phantom and lamb lungs to detect water contents as small as 1 mL. The system was also shown to be successful in monitoring progress of fluid accumulation as the calculated intensity of the scattered field varies linearly with the amount of accumulated fluid. Preclinical human trials were also conducted on a number of volunteers to investigate the possibility of defining a preliminary range for healthy people. The initial results show an encouraging trend in maximum field intensity that varies by only 10 percent for people with different body dimensions. Overall, considering the detection limitations and safety concerns of the conventional devices in quantifying and monitoring small amounts of fluid, which is the sign of various diseases, the proposed platform was attested as a reliable, convenient and practical prototype that can be verified in future clinical trials.

## Additional Information

**How to cite this article**: Rezaeieh, S. A. *et al.* Feasibility of Using Wideband Microwave System for Non-Invasive Detection and Monitoring of Pulmonary Oedema. *Sci. Rep.* **5**, 14047; doi: 10.1038/srep14047 (2015).

## Figures and Tables

**Table 1:** Geometrical details in (mm) of the optimum design.

*l*_*1*_	*w*_*1*_	*l*_*m*_	*l*_*m2*_	*l*_*l1*_	*l*_*l3*_	*l*_*l5*_	*w*_*l1*_	*w*_*l3*_	*w*_*l5*_
8	24	16	42	10	2	20	20	10	15
*l*_*2*_	*w*_*2*_	*l*_*m1*_	*l*_*p*_	*l*_*l2*_	*l*_*l4*_	*l*_*l6*_	*w*_*l2*_	*w*_*l4*_	*w*_*l6*_
30	5	44	58	4	4	4	50	15	30
